# NFE2L3 as a Potential Functional Gene Regulating Immune Microenvironment in Human Kidney Cancer

**DOI:** 10.1155/2022/9085186

**Published:** 2022-10-26

**Authors:** Qian Zhang, Donge Tang, Aiyun Zha, Jingquan He, Dandan Li, Yumei Chen, Wanxia Cai, Jian Dai, Shaodong Luan, Lianghong Yin, Wei Zhang, Yong Dai

**Affiliations:** ^1^Clinical Medical Research Center, The Second Clinical Medical College of Jinan University, Shenzhen People's Hospital, Shenzhen 518020, China; ^2^Institute of Nephrology, The First Affiliated Hospital of Jinan University, Guangzhou 510632, China; ^3^Department of Biomedical Engineering, Jinan University, Guangzhou 510630, China; ^4^Department of Nephrology, Shenzhen Longhua District Central Hospital, Shenzhen 518110, China; ^5^Institute of Nephrology and Blood Purification, Jinan University, Guangzhou 510632, China

## Abstract

With the increasing incidence and mortality of renal cancer, it is pressing to find new biomarkers and drug targets for diagnosis and treatment. However, as one negative upstream regulator of p53, the prognostic and immunological role of NFE2L3 in renal cancer is still barely known. We investigated the expression, prognostic value, and relevant pathways of NFE2L3 using the datasets from public databases, including The Cancer Genome Atlas Program (TCGA), Genotype-Tissue Expression (GTEx), Cancer Cell Line Encyclopedia (CCLE), and UALCAN. Furthermore, we analyzed the relationship between NFE2L3 expression and the immune microenvironment using distinct methods. We found that NFE2L3 was higher expressed in kidney renal clear cell carcinoma (KIRC) and kidney renal papillary cell carcinoma (KIRP) tissues than adjacent normal tissues. Additionally, we identified NFE2L3 as one survival-related factor for KIRC and KIRP. The enrichment analyses revealed that NFE2L3 was associated with a variety of immune-relevant pathways in KIRC and related to the infiltration ratios of 17 types of immune cells in KIRC patients. Ultimately, we demonstrated nine significantly enriched mutations, such as TP53 and MET, in NFE2L3-expression-changing groups. The elevated expression of NFE2L3 in renal cancerous tissues versus normal tissues is associated with poor outcomes in patients. Besides, NFE2L3 has a role in the regulation of the immune microenvironment in renal cancer patients. The findings of our study provide a potential prognostic biomarker and a new drug target for renal cancer.

## 1. Introduction

Renal cell carcinoma (RCC) is a malignant tumor derived from proximal renal tubular epithelial cells, which account for 90% of all kidney cancer cases [1]. The incidence of RCC comes after prostate cancer and bladder cancer in all urinary cancers [2]. Among the common subtypes of RCC, kidney renal clear cell carcinoma (KIRC) accounts for 75-80% of the total cases [3], followed by kidney renal papillary cell carcinoma (KIRP) [4] and kidney chromophobe (KICH) [5]. In 2018, there were 63,000 new RCC cases in the United States [6]. Most RCC cases are diagnosed in an advanced stage with distal metastases, and a quarter of the cases are fatal [7–9].

Currently, immune checkpoint drugs are the most common treatment for metastatic RCC, but they work for only a small percentage of RCC patients [10]. Latest researches have demonstrated that RCC patients may achieve more benefits from the combination of several immune checkpoint inhibitors than a single inhibitor [11]. Consequently, it is especially important to search for new immune therapeutic targets developed by drugs, used alone or in combination.

Nuclear factor erythroid-derived 2-like 3 (NFE2L3), one member of the cap‘n'collar basic region leucine zipper transcription factor family, is involved in several important cellular processes, such as stress response and signal transduction [12]. Several researches have revealed that NFE2L3 is involved in the carcinogenesis of pancreatic cancer and liver cancer, and the increased expression is associated with poorer prognosis of patients [13–15]. As one upstream negative regulator of p53, NFE2L3 induces cell growth via stimulating the degradation of p53 in colon cancer cells [16, 17]. Because TP53 is one core suppressor gene in cells [18], NFE2L3 may play an important role in tumorigenesis as well. However, there are only a few reports of NFE2L3 in RCC [19], and its relationship with prognosis and underlying mechanisms in RCC tumorigenesis are still not clear.

In our study, we investigated NFE2L3 expression in RCC tissues versus normal adjacent tissues and analyzed its prognostic value based on datasets from the public database. Besides, we analyzed the relevant pathways of NFE2L3 in RCC. Furthermore, we explored the role of NFE2L3 in the immune microenvironment. Ultimately, we analyzed the mutations correlated with NFE2L3 expression in RCC patients.

## 2. Methods

### 2.1. TIMER

TIMER (http://timer.cistrome.org/) is a comprehensive resource database that systematically analyzes the immune infiltration levels of different cancers [20]. Differential expressions of NFE2L3 between tumorous tissues and adjacent normal tissues were noted in 27 types of tumors based on datasets from The Cancer Genome Atlas Program (TCGA) and Genotype-Tissue Expression (GTEx) using the DiffExp module. The statistical significance of differential expression was evaluated by the Wilcoxon test, and a statistically significant *P* value was set as 0.05.

### 2.2. UALCAN

UALCAN (http://ualcan.path.uab.edu/index.html) is an online mining and analyzing database for in-depth analysis of transcriptomic data from TCGA [21]. UALCAN was used to analyze the expression of NFE2L3 and the relationship between NFE2L3 and several clinicopathological parameters of RCC patients, including gender, tumor stage, lymph node metastasis status, age, and race.

### 2.3. Kaplan-Meier Plotter

The Kaplan-Meier Plotter database (http://kmplot.com/analysis/index.php?p=background) is constructed based on the gene chip and RNA-seq data from other public databases [22], such as Gene Expression Omnibus (GEO) and TCGA. All patient samples are divided into two groups based on the median expression of genes (high expression and low expression). In the two groups, the disease-free interval rate (DFI), disease-specific survival rate (DSS), progression-free survival rate (PFS), and overall survival rate (OS) of RCC patients were analyzed. The confidence interval (CI) was set as 95%. The “Pan-cancer RNA-seq” function was used to analyze the correlation between NFE2L3 expression and the survival rates of RCC patients with high immune cell infiltration or low immune cell infiltration.

### 2.4. Enrichment Analysis

Gene Ontology (GO), Kyoto Encyclopedia of Genes and Genomes (KEGG), and Gene Set Enrichment Analysis (GSEA) analyses were used to explore the biological functions of NFE2L3 in RCC. GO analysis is a powerful bioinformatic approach to determining the biological process (BP), cellular component (CC), and molecular function (MF) that NFE2L3 involves in. GSEA was used to investigate the potential active or inactive pathways that NFE2L3 participates in.

### 2.5. Linkedomics

The Linkedomics database (http://www.linkedomics.org/login.php) was utilized to obtain the top 50 genes with positive or negative expression correlations with NFE2L3 in RCC tissues [23].

### 2.6. Bioinformatics

R package “survival v.2.4.2” was used for the survival analysis and progression-free survival analysis (PFS). The nomogram survival maps were constructed using R packages “survival” and “rms.” The R package “forest plot” was used to visualize the results of multivariate Cox regression of NFE2L3. The *P* values are two-sided, and a *P* value < 0.05 is considered statistically significant.

### 2.7. Software

Based on a valid leukocyte gene signature matrix containing 547 genes and 22 human immune cell subgroups, CIBERSORT (https://cibersort.stanford.edu/) was used to establish a computing resource to characterize the immune cell composition of RCC [24]. The correlation between NFE2L3 expression and infiltrated immune cell ratios was calculated by Pearson analysis. A *P* value < 0.05 was considered statistically significant.

### 2.8. Statistical Analyses

The hazard ratio (HR) of NFE2L3 in RCC was calculated using univariate Cox regression [25]. The independent prognostic factor was determined using multivariable Cox regression [26].

## 3. Results

### 3.1. Increased Expression of NFE2L3 in RCC Patients

**Figure 1:**
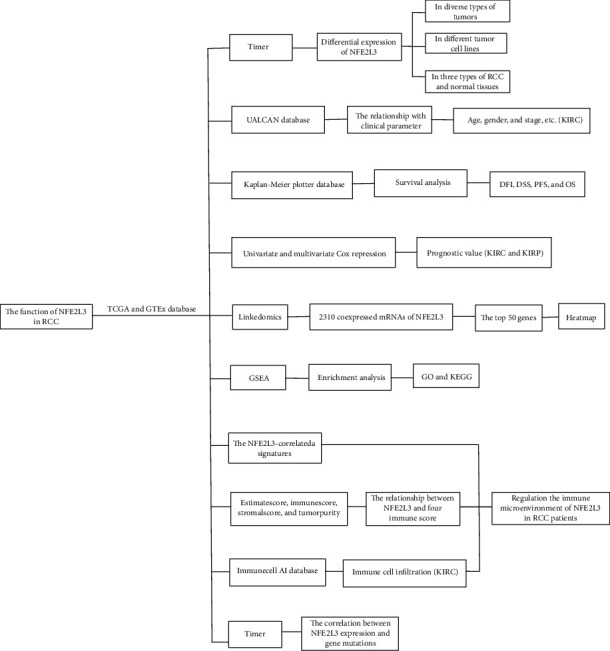
The roadmap of our study.

**Figure 2:**
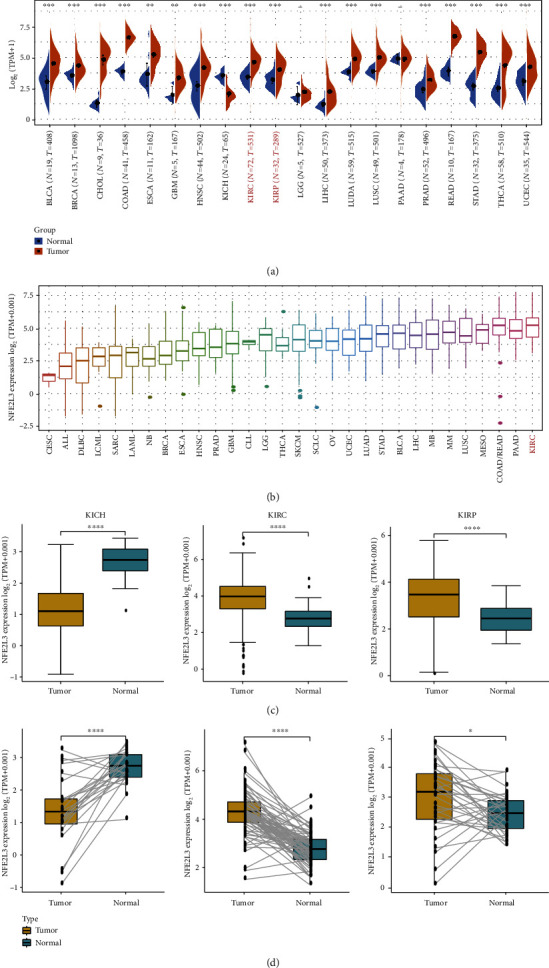
Expression of NFE2L3 in renal cell carcinoma: (a) NFE2L3 expression in diverse types of tumors as analyzed using TIMER database; (b) NFE2L3 expression in different tumor cell lines; (c) NFE2L3 expression in three subtypes of RCC and adjacent normal tissues; (d) expression of NFE2L3 in paired KICH, KIRC, and KIRP tissues and adjacent normal tissues, respectively. ^∗^*P* < 0.05,  ^∗∗^*P* < 0.01,  ^∗∗∗^*P* < 0.001, and^∗∗∗∗^*P* < 0.0001.

First, we showed the roadmap of our study (Figure 1). To explore the role of NFE2L3 in RCC, we first analyzed the expression of NFE2L3 in various cancers using the datasets from TCGA database.  Figure 2(a) reveals an increased expression of NFE2L3 in 25 types of cancers, including bladder urothelial carcinoma (BLCA), breast invasive carcinoma (BRCA), cervical squamous cell carcinoma and endocervical adenocarcinoma (CESC), cholangiocarcinoma (CHOL), colon adenocarcinoma (COAD), esophageal carcinoma (ESCA), glioblastoma multiforme (GBM), head and neck squamous cell carcinoma (HNSC), acute myeloid leukemia (LAML), brain low-grade glioma (LGG), liver hepatocellular carcinoma (LIHC), lung adenocarcinoma (LUAD), lung squamous cell carcinoma (LUSC), ovarian serous cystadenocarcinoma (OV), pancreatic adenocarcinoma (PAAD), pheochromocytoma and paraganglioma (PRAD), rectum adenocarcinoma (READ), skin cutaneous melanoma (SKCM), stomach adenocarcinoma (STAD), testicular germ cell tumors (TGCT), thyroid carcinoma (THCA), uterine corpus endometrial carcinoma (UCEC), uterine carcinosarcoma (UCS), KIRC, and KIRP tissues versus the normal adjacent tissues. Additionally, using the datasets from the Cancer Cell Line Encyclopedia (CCLE) database, we compared the NFE2L3 expression among multiple cancer cell lines and found that NFE2L3 was higher expressed in sarcoma (SARC), LGG, and KIRC cell lines, while lower expressed in LUSC, PRAD, and HNSC cell lines (Figures 2(b)). In particular, we displayed the expression of NFE2L3 in cancerous tissues versus normal tissues of KICH, KIRC, and KIPR patients using the datasets from TCGA. As shown in Figure 2(c), an elevated expression of NFE2L3 was observed in KIRC and KIRP tissues compared to normal tissues. Notably, an increased expression of NFE2L3 in KIRC and KIRP was also observed in the paired tumorous tissues versus adjacent normal tissues from TCGA (Figure 2(d)). The above findings clarified that NFE2L3 expression was upregulated in RCC patients and NFE2L3 might play a role in RCC progression.

### 3.2. NFE2L3 Was Potentially Associated with Cancer Progression of KIRC

**Figure 3:**
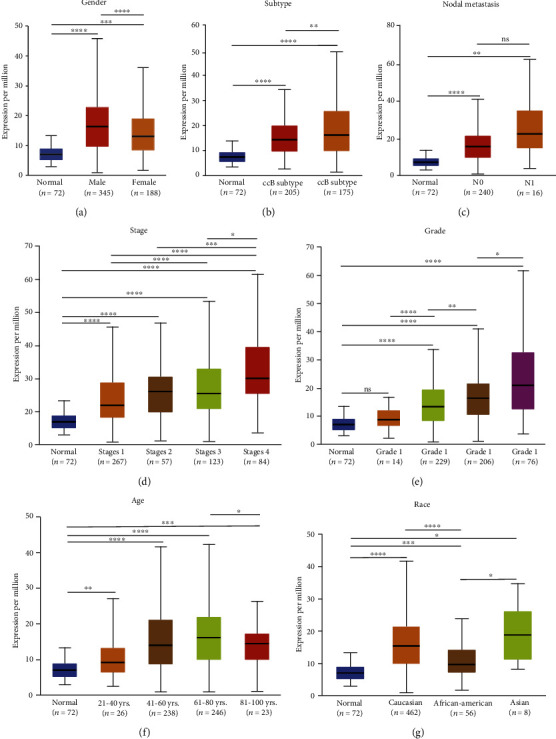
Box plots showing NFE2L3 expression among different groups of KIRC patients based on clinical parameters as analyzed using the UALCAN database. Analysis was shown for (a) patient's gender, (b) subtype, (c) nodal metastasis, (d) cancer stage, (e) tumor grade, (f) patients' age, and (g) patients' race. N0: no regional lymph node metastasis; N1: metastases in 1 to 3 axillary lymph nodes. ^∗^*P* < 0.05,  ^∗∗^*P* < 0.01,  ^∗∗∗^*P* < 0.001, and^∗∗∗∗^*P* < 0.0001.

Since KIRC accounts for the largest proportion of RCC, we chose KIRC as a representative model to demonstrate the correlation between the NFE2L3 expression and some clinical indicators. Using the datasets from the UALCAN database, we found that the NFE2L3 expression was significantly increased in male KIRC patients compared to females (*P* < 0.001) (Figure 3(a)). NFE2L3 was higher expressed in the B subtype of KIRC than in the A subtype of KIRC (*P* < 0.01) (Figure 3(b)). Moreover, the NFE2L3 expression was gradually increased with N stage, stage, and grade (Figures 3(c)–3(e)). For example, a significant elevation was seen in grade 2 versus grade1 (*P* < 0.0001), grade 3 versus grade2 (*P* < 0.01), and grade 4 versus grade 3 (*P* < 0.05) (Figure 3(e)). Furthermore, the NFE2L3 expression was significantly elevated in cancerous tissues of KIRC patients with age, ranging from 21 to 40 years, 41 to 60 years, and 61 to 80 years (Figure 3(f)). As shown in Figure 3(g), the highest expression of NFE2L3 was prominently observed in Caucasian and Asian KIRC samples. Herein, these results manifested a potential relevance between NFE2L3 expression and tumor progression.

### 3.3. The NFE2L3 Expression Was Correlated with the Survival Rates of RCC Patients

**Figure 4:**
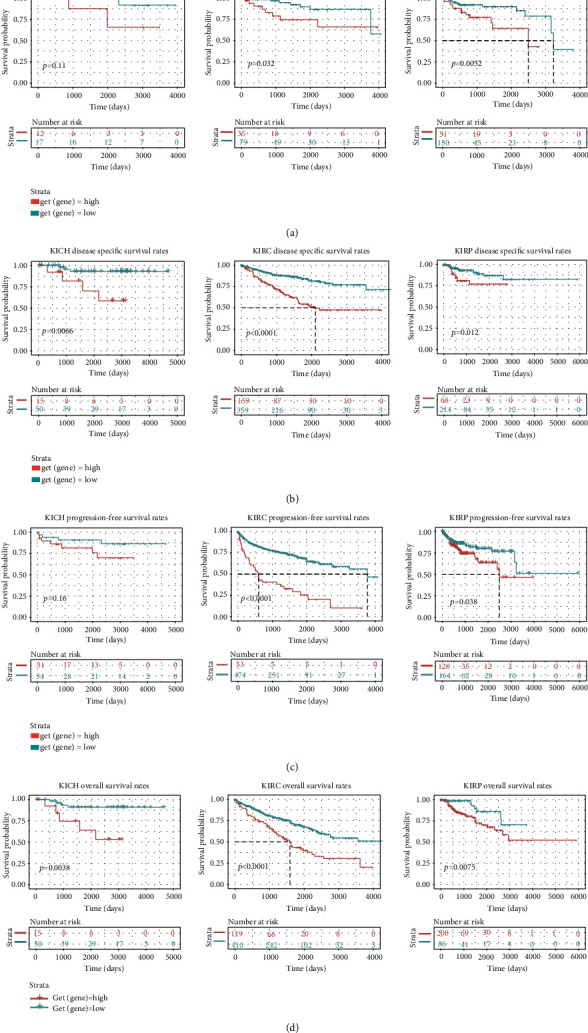
NFE2L3 expression was correlated with the survival of RCC patients. Survival curves were shown for (a) DFI, (b) DSS, (c) PFS, (d) and OS of three subtypes of RCC as analyzed using the Kaplan-Meier plotter database.

**Figure 5:**
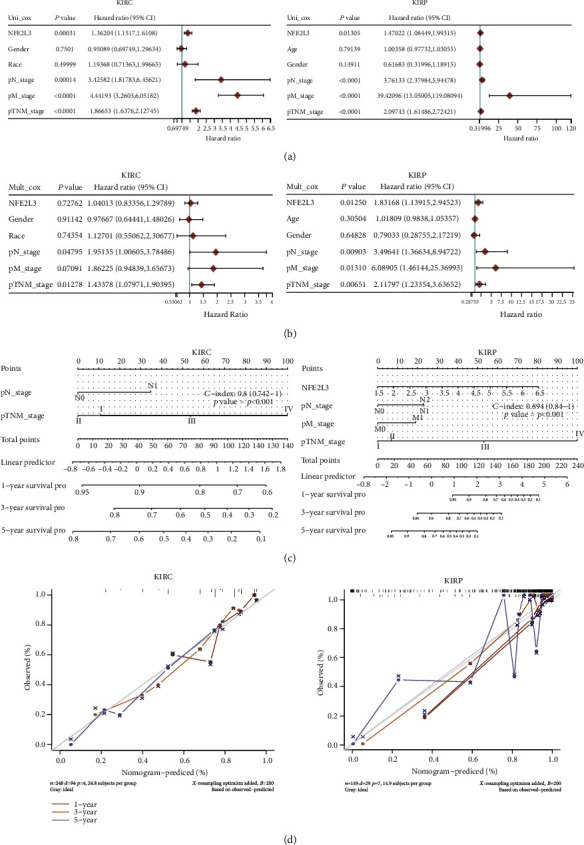
The prognostic value of NFE2L3: (a) a forest plot showing the HR of NFE2L3 expression in KIRC and KIRP patients as investigated using univariate Cox regression; (b) a forest plot showing NFE2L3 as a potential independent prognostic factor for KIRC and KIRP as investigated using multivariable Cox regression; (c, d) colliographs showed the use of NFE2L3 in the prognostic risk scoring of KIRC and KIRP.

Subsequently, we tested the prognostic value of NFE2L3 in RCC patients. For KIRC, the patients with higher NFE2L3 expression exhibited a poorer DFI (*P* < 0.05), DSS (*P* < 0.0001), PFS (*P* < 0.0001), and OS (*P* < 0.0001) (Figures 4(a)–4(d)), and in KIRP patients, the higher NFE2L3 expression was also associated with a poorer DFI (*P* < 0.01), DSS (*P* < 0.05), PFS (*P* < 0.05), and OS (*P* < 0.01) (Figures 4(a)–4(d)). Furthermore, the univariate Cox regression indicated that NFE2L3 was both a risk factor for KIRC (*P* < 0.001) and KIRP (*P* < 0.05) patients (Figure 5(a)), and the multivariate Cox regression elucidated that NFE2L3 was one independent prognostic factor in KIRP patients (*P* < 0.05) (Figure 4(b)). The diagram in Figures 5(c) and 5(d) indicated that NFE2L3 might be used in combination with other clinical indicators to predict the outcomes of KIRC and KIPR patients.

### 3.4. NFE2L3 Was One Potential Regulatory Factor of the Immune Microenvironment of RCC Patients

**Figure 6:**
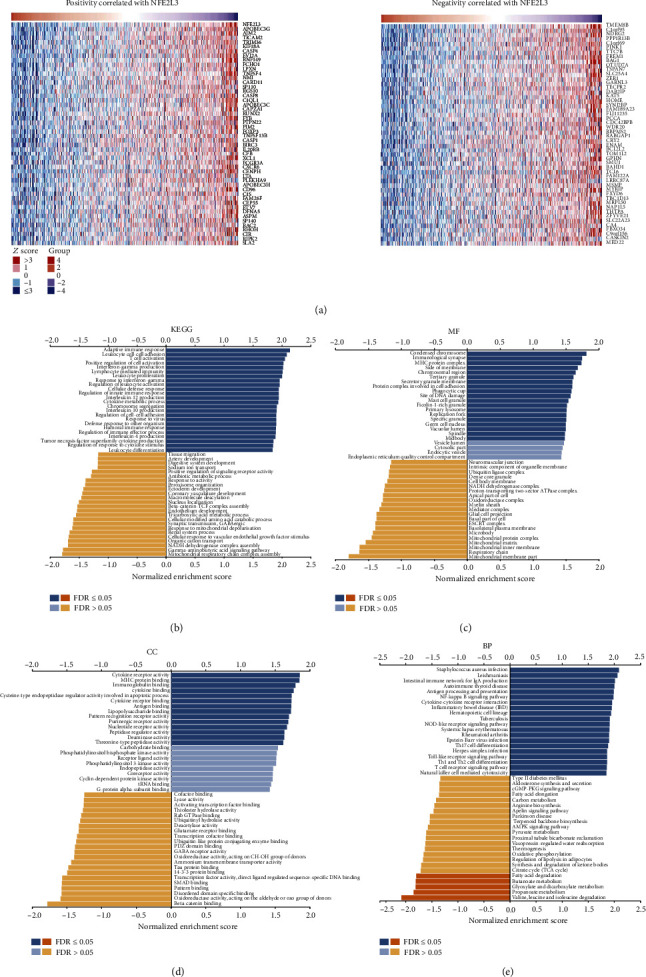
NFE2L3-relevant biological activities and pathways in KIRC: (a) a heat map showing the top 50 genes positively or negatively correlated with NFE2L3 in KIRC. Based on the analysis of NFE2L3-coexpressed genes, the top 20 enrichment terms of (b) KEGG, (c) MF, (d) CC, and (e) BP.

As KIRC accounts for the largest proportion of RCC, we chose KIRC as a representative model to clarify the mechanisms involved in the function of NFE2L3 in KIRC. Firstly, we analyzed the coexpressed mRNAs of NFE2L3 using the datasets from Linkedomics. As a result, we obtained 2310 NFE2L3 coexpressed genes (Supplementary Table S1). The top 50 genes positively or negatively correlated with NFE2L3 are displayed in Figure 6(a). To explore the NFE2L3-related pathways, we used GSEA to note the top 20 significant terms of KEGG, MF, CC, and BP. In terms of KEGG terms, the relevant pathways of NFE2L3 are mainly enriched in immune-related processes, such as adaptive immune response, leukocyte cell-cell adhesion, T cell activation, and positive regulation of cell activation (Figure 6(b)). In MF terms, NFE2L3 is chiefly enriched in condensed chromosomes, immunological synapses, MHC protein complexes, and protein complexes involved in cell adhesion (Figure 6(c)). As terms of CC, the cellular components of NFE2L3 were primarily enriched in cytokine receptor activity, MHC protein binding, immunoglobulin binding, and cytokine binding (Figure 6(d)). In BP terms, Figure 6(e) exhibited that the biological function of NFE2L3 was mainly enriched in the nuclear factor-kappa B (NF-*κ*B) signaling pathway, autoimmune thyroid disease, cytokine-cytokine receptor interaction, and NOD-like receptor signaling pathway. These results indicated that the functions of NFE2L3 in KIRC were possibly closely relevant to the immune microenvironment.

**Figure 7:**
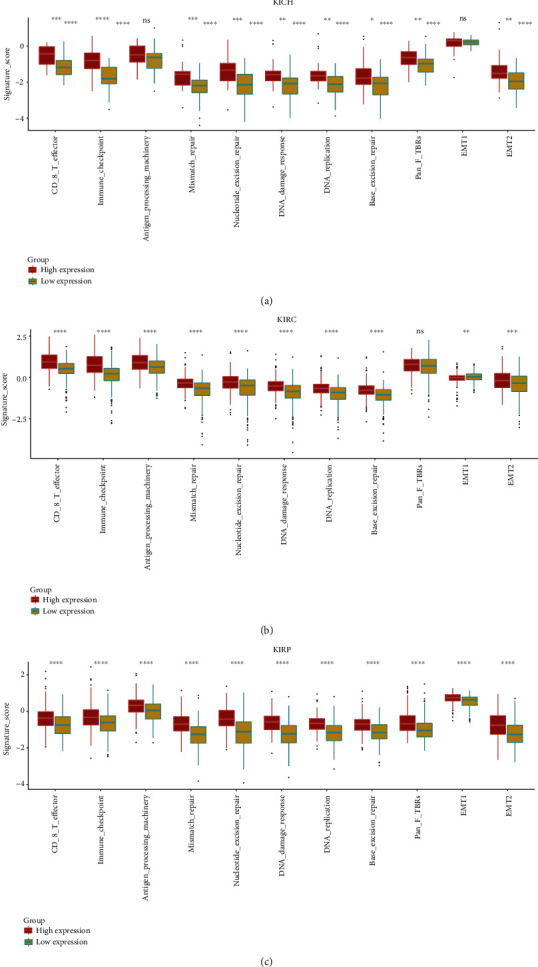
The NFE2L3-correlated signatures in three subtypes of RCC. The correlation between NFE2L3 expression and multiple signatures in (a) KICH, (b) KIRC, and (c) KIRP. ^∗^*P* < 0.05,  ^∗∗^*P* < 0.01,  ^∗∗∗^*P* < 0.001, and^∗∗∗∗^*P* < 0.0001.

Next, we estimated the NFE2L3-correlated immune signatures in different subtypes of RCC. We found that the high expression of NFE2L3 was correlated with the immune signatures including immune checkpoint, effector CD8 T cells, and antigen processing machinery in KICH and KIRP (Figures 7(a)–7(c)). Meanwhile, NFE2L3 was positively correlated with some other signatures, namely, DNA replication, mismatch repair, DNA damage, type 1 epithelial-mesenchymal transition (EMT1), and type 2 epithelial-mesenchymal transition (EMT2) in KICH, KIRC, and KIRP patients (Figures 7(a)–7(c)). These results further suggested that the functions of NFE2L3 in RCC might be related to immune regulation.

**Figure 8:**
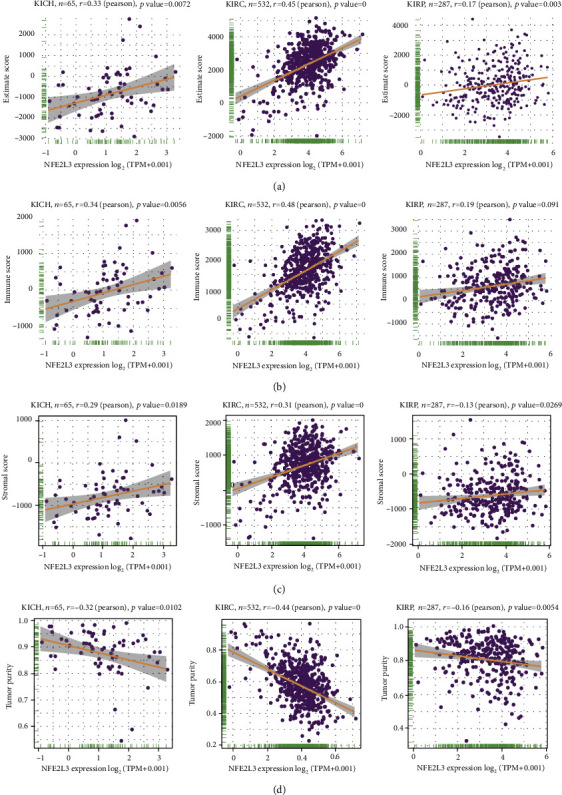
The correlation between NFE2L3 expression and diverse types of immune scores. The correlation between NFE2L3 expression and (a) estimate score, (b) immune score, (c) stromal score, and (d) tumor purity.

**Figure 9:**
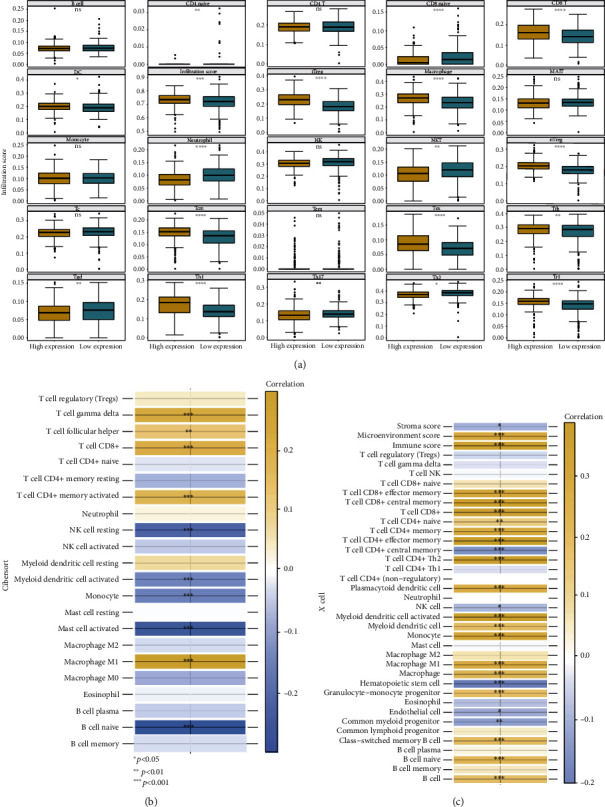
The correlation between NFE2L3 expression and infiltration levels of various immune cells: (a) the relationship between NFE2L3 expression and the infiltration ratios of various immune cells in KIRC as analyzed using ImmuCellAi; (b) the relationship between NFE2L3 expression and the infiltration ratios of various immune cells as analyzed using CIBERSORT algorithm; (c) the relationship between NFE2L3 expression and the infiltration ratios of various immune cells as analyzed using Xcell algorithm. ^∗^*P* < 0.05,  ^∗∗^*P* < 0.01,  ^∗∗∗^*P* < 0.001, and^∗∗∗∗^*P* < 0.0001.

Consequently, to deepen and understand the role of NFE2L3 in the immune microenvironment in RCC patients, we investigated the association between four types of immune scores and NFE2L3 expression in three subtypes of RCC. As shown in Figures 8(a)–8(d), the expression of NFE2L3 in KICH, KIRC, and KIRP was significantly correlated with immune score, ESTIMATE score, stromal score, and tumor purity. Additionally, since KIRC is the most common subtype of RCC, we select KIRC as a representative model to illustrate the relationship between NFE2L3 and immune infiltration in RCC. We investigated the correlation between NFE2L3 expression and infiltration ratios of various immune cells. We found that the NFE2L3 expression was positively associated with the infiltration ratios of 17 types of immune cells, such as naive CD4+ cells, CD4 T+ cells, naive CD8+ cells, CD8+ T cells, dendritic cells, neutrophils, type 1 helper T (Th1) cells, type 17 helper T (Th17) cells, and type 1 regulatory T (Tr1) cells as analyzed by ImmuCellAi (Figure 9(a)). Using the analysis tool CIBERSORT, we discovered an association between the NFE2L3 expression and the infiltration ratios of 10 types of immune cells, including gamma delta T cells, follicular helper T cells, CD8+ T cells, activated memory CD4+ T cells, resting natural killer cells, activated myeloid dendritic cells, monocytes, activated mass cells, M1 macrophages, and naive B cells (Figure 9(b)). Besides, we also observed that NEF2L3 expression was strongly correlated with the infiltration ratios of 22 types of immune cells as analyzed using Xcell, including effector memory CD8+ T cells, central memory CD8+ T cells, CD8+ T cells, naive CD4+ T cells, memory CD4+ T cells, effector memory CD4+ T cells, central memory CD4+ T cells, type 2 helper T cells, plasmacytoid dendritic cells, natural killer (NK) cells, activated myeloid dendritic cells, myeloid dendritic cells, monocytes, M1 macrophages, macrophages, hematopoietic stem cells, granulocyte-monocyte progenitors, endothelial cells, common myeloid progenitors, class-switched memory B cells, naive B cells, and B cells (Figure 9(c)). These results revealed that NFE2L3 was one potential regulatory factor of the immune microenvironment of RCC patients.

### 3.5. NFE2L3 May Be Associated with Some Gene Mutations in the Development of Kidney Cancer

**Figure 10:**
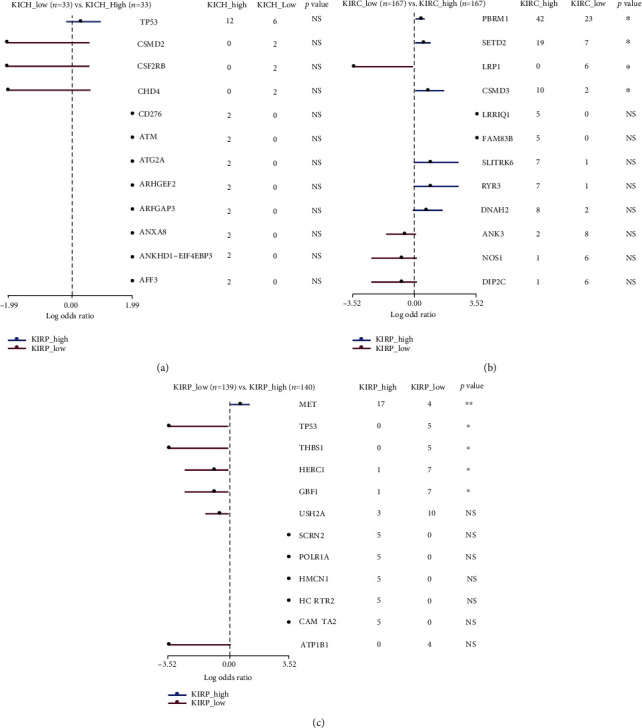
The correlation between NFE2L3 expression and frequency of gene mutations in three subtypes of RCC. The correlation between NFE2L3 expression and frequencies of gene mutations in (a) KICH, (b) KIRC, and (c) KIRP.

Finally, we explored the potential relationship between several common mutation genes and the occurrence of kidney cancer. There was no mutated gene relevant to the development of KICH no matter in the NFE2L3 high expression group or the low expression group (Figure 10(a)). It may be related to the lower degree of malignancy of KICH. As showed in Figure 10(b), in KIRC, NFE2L3 high expression was associated with the mutation of PBRM1, SETD2, and CSMD3. In KIRP, NFE2L3 high expression was correlated with MET mutation (Figure 10(c)). This information indicated that in the development of kidney cancer, the NFE2L3 expression was related to distinct gene mutations.

## 4. Discussion

In our study, we discovered the elevated expression of NFE2L3 in KIRC and KIRP tissues versus normal adjacent tissues, identified NFE2L3 as one survival-relevant gene for KIRC and KIRP, and uncovered a potential regulatory role of NFE2L3 in the immune microenvironment in RCC patients. These results disclosed a new vision of NFE2L3 as one promising prognosis biomarker and functional gene in RCC patients.

The earlier report by Wang et al. [19] has reported 3 genes (LAT, HOXD3, and NFE2L3) as a potential prognostic biomarker for kidney renal clear cell carcinoma (KIRC). They depicted the effect of the three DNA methylation-driven gene (LAT, HOXD3, and NFE2L3) expression on survival rate and as independent prognostic factors of KIRC patients. Correspondingly, the main point of our paper is to focus on the regulatory role of NFE2L3 in the immune microenvironment of kidney cancer. Excepting for the immunological findings, we revealed for the first time a positive association between the NFE2L3 expression and the frequency of MET, PBRM1, and SETD mutations. Meanwhile, other results of our study also expanded and complemented the earlier report [19].

As an important regulator of cellular stress response, NFE2L3 is abundantly expressed in various organs, such as the kidney, pancreas, heart, lung, liver, and brain [27, 28]. In our study, we found that NFE2L3 had a potential relationship with the NF-*κ*B pathway (Figure 6). In colorectal cancer, NFE2L3 regulates the growth of CRC via the NF-*κ*B pathway [29]. Therefore, our results are consistent with the previous researches [19]. As is well known, NF-*κ*B has a critical role in many cancer-relevant processes, such as inflammation [30, 31]. Hence, integrating our results with the previous findings, we speculate that NFE2L3 may be functional in the immune microenvironment in RCC patients depending at least in part on the NF-*κ*B pathway.

Nowadays, the treatments for metastatic RCC patients have gone from interferon *α* and IL2 to immune checkpoint drugs [32, 33]. Due to the obvious side effects and fewer complete remission ratios of patients treated with interferon *α* and IL2, monoclonal antibodies against the immune checkpoints have been used in the treatment of metastatic renal cancer in recent years [34]. However, the drug resistance of immune checkpoint inhibitors leads tumor patients to only benefit from the treatment for a limited time [35]. Therefore, it is necessary to continue developing new targets for immune therapy. In our study, NFE2L3 is revealed as one probable regulatory gene for the immune microenvironment. Meanwhile, considering that NFE2L3 may be an upstream regulator of NF-*κ*B in RCC [19], it is of great significance to further study the mechanism of NFE2L3 to explore its possibility as a drug target.

Tumor microenvironment (TME) is regarded as the cellular environment around tumors, in which exist blood vessels, lymphocytes, immune cells, fibroblasts, extracellular matrix, and other components [36]. The immune cells from TME influence the initiation and progression of cancers [37]. For example, infiltrated CD8+ T cells in colon cancer frequently have an antitumor effect [38]. More regulatory T cells (Tregs) in tumor tissues always indicate a poor prognosis for tumor patients [39]. KIRC is a kind of malignant tumor accompanied by high infiltration of immune cells and is one of the earliest malignant tumors treated by immune drugs as well [40]. In this study, we also revealed that the infiltration ratios of CD8+ T cells, Tregs, and macrophages were associated with the NFE2L3 expression in KIRC patients. This finding further supports that NFE2L3 may be a promising immune target in RCC patients.

Finally, we demonstrated that the mutated METS were strongly enriched in the NFE2L3-high-expression KIRP patients in our study. This result indicates that the influence of NFE2L3 on the occurrence and development of KIRP may be related to the MET mutations. Encoded by MET, c-Met is a type of transmembrane receptor tyrosine kinase. In fact, c-Met is frequently activated in type I KIRP, thereby stimulating c-Met/HGF signaling and promoting many biological processes, such as cell proliferation, angiogenesis, and malignant transformation [41].

## Data Availability

The original contributions presented in the study are included in the article/Supplementary Materials; further inquiries can be directed to the corresponding authors.

## Supplementary Materials

Supplementary Materials Supplementary Table S1: the 2310 NFE2L3-coexpressed genes.
