# Supplementary material for: NFE2L3 as a Potential Functional Gene Regulating Immune Microenvironment in Human Kidney Cancer
Source: Biomed Res Int. 2022 Oct 26;2022:9085186. doi: 10.1155/2022/9085186 (PMC9629961; doi:10.1155/2022/9085186)
Supplement: Supplementary Materials — Supplementary Table S1: the 2310 NFE2L3-coexpressed genes. [file 9085186.f1.pdf]

Query	Statistic	P-value	FDR (BH)	Event_	Event_
ABCA8	-0.388189065	1.30E-20	3.03E-19	533	530
ABCC2	0.380999982	7.39E-20	1.56E-18	533	533
ABCC3	0.300271115	1.44E-12	1.26E-11	533	533
ABCC6P	0.306378662	4.78E-13	4.46E-12	533	521
ABCD2	0.357716773	1.56E-17	2.48E-16	533	531
ABCG2	-0.35243451	4.94E-17	7.43E-16	533	533
ABL1	-0.397569748	1.25E-21	3.32E-20	533	533
ACAA2	-0.339606157	7.45E-16	9.74E-15	533	533
ACAP1	0.423159164	1.44E-24	5.54E-23	533	532
ACAT1	-0.38483072	2.94E-20	6.53E-19	533	533
ACER3	0.37782583	1.57E-19	3.18E-18	533	533
ACOT1	-0.306415927	4.75E-13	4.43E-12	533	533
ACP2	0.32976467	5.50E-15	6.54E-14	533	533
ACRBP	0.440104281	1.17E-26	5.90E-25	533	533
ACSL1	-0.361977654	6.04E-18	1.01E-16	533	533
ACSL5	0.317475727	6.03E-14	6.25E-13	533	533
ACTBL2	0.311034019	2.03E-13	1.98E-12	533	318
ACTR10	-0.395163065	2.30E-21	5.95E-20	533	533
ACTR3	0.343160827	3.56E-16	4.84E-15	533	533
ACVR2A	-0.370873327	7.99E-19	1.48E-17	533	533
ADAM12	0.310808845	2.11E-13	2.06E-12	533	533
ADAM28	0.412065462	2.91E-23	9.45E-22	533	533
ADAM6	0.346902896	1.62E-16	2.27E-15	533	533
ADAM8	0.409632385	5.54E-23	1.74E-21	533	533
ADAMD	0.397967759	1.13E-21	3.02E-20	533	530
ADAMTS	-0.348242282	1.22E-16	1.74E-15	533	523
ADAMTS	0.381642361	6.34E-20	1.35E-18	533	533
ADAP2	0.397237546	1.36E-21	3.59E-20	533	533
ADA	0.358538704	1.30E-17	2.09E-16	533	533
ADCY1	-0.328969155	6.44E-15	7.60E-14	533	533
ADCY5	-0.365130777	2.97E-18	5.11E-17	533	532
ADCY7	0.4479597	1.15E-27	6.54E-26	533	533
ADH5	-0.363516278	4.28E-18	7.25E-17	533	533
ADORA3	0.400259925	6.31E-22	1.73E-20	533	533
ADPGK	0.382904863	4.68E-20	1.01E-18	533	533
ADRA2B	-0.337613473	1.12E-15	1.44E-14	533	533
ADRBK2	0.355628983	2.46E-17	3.85E-16	533	533
AGBL2	0.331078158	4.23E-15	5.08E-14	533	529
AGPAT9	-0.381164977	7.11E-20	1.50E-18	533	533
AGTR1	-0.387413664	1.57E-20	3.62E-19	533	533
AIF1	0.469598305	1.38E-30	1.22E-28	533	533
AIM1L	0.446640875	1.70E-27	9.46E-26	533	511
AIM1	0.385285135	2.63E-20	5.93E-19	533	533
AIM2	0.623009759	1.26E-58	8.45E-55	533	530
AK1	-0.361651771	6.50E-18	1.08E-16	533	533
AK3	-0.336650635	1.37E-15	1.73E-14	533	533
AKAP5	0.355658468	2.45E-17	3.82E-16	533	533
AKNA	0.35109841	6.59E-17	9.69E-16	533	533
AKR1C1	-0.325737339	1.22E-14	1.39E-13	533	533
ALAD	-0.352375957	5.00E-17	7.50E-16	533	533
ALDH18	0.384120533	3.49E-20	7.68E-19	533	533
ALDH1B	-0.312511016	1.54E-13	1.53E-12	533	533
ALDH1L	-0.446625885	1.71E-27	9.48E-26	533	533
ALDH3A	-0.34162788	4.90E-16	6.57E-15	533	533
ALDH6A	-0.405531056	1.62E-22	4.81E-21	533	533
ALG2	-0.387166814	1.66E-20	3.83E-19	533	533
ALG6	0.363450356	4.34E-18	7.36E-17	533	533
ALKBH1	-0.310182394	2.38E-13	2.30E-12	533	533
ALOX5A	0.317298259	6.24E-14	6.45E-13	533	533
ALOX5	0.385021921	2.81E-20	6.27E-19	533	533
ALPK1	0.335809934	1.62E-15	2.03E-14	533	533
ALPK3	-0.323301275	1.96E-14	2.17E-13	533	533
AMICA1	0.310780452	2.13E-13	2.07E-12	533	533
AMMEC	0.331282651	4.06E-15	4.89E-14	533	533
AMOTL1	-0.312175973	1.64E-13	1.62E-12	533	533
AMPD2	0.358158139	1.41E-17	2.26E-16	533	533
ANAPC2	-0.337309859	1.20E-15	1.52E-14	533	533
ANAPC5	0.333524591	2.58E-15	3.17E-14	533	533
ANKLE2	0.347994043	1.28E-16	1.83E-15	533	533
ANKRD2	0.426763445	5.29E-25	2.15E-23	533	533
ANKRD5	-0.316508951	7.25E-14	7.42E-13	533	531
ANKRD5	0.500432638	3.98E-35	7.06E-33	533	533
ANKS1A	-0.344735954	2.56E-16	3.52E-15	533	533
ANLN	0.498779376	7.16E-35	1.19E-32	533	532
ANO3	-0.312271209	1.61E-13	1.59E-12	533	528
ANXA2	0.311483659	1.87E-13	1.83E-12	533	533
ANXA8L	0.353001132	4.37E-17	6.63E-16	533	378
AOAH	0.432173275	1.15E-25	5.06E-24	533	533
AP1S3	0.358534492	1.30E-17	2.09E-16	533	533
AP2A2	-0.329466899	5.83E-15	6.92E-14	533	533
AP3S1	0.435472027	4.48E-26	2.08E-24	533	533
AP4M1	0.420976632	2.62E-24	9.69E-23	533	533
AP4S1	-0.361747654	6.36E-18	1.06E-16	533	533
APAF1	0.37952262	1.05E-19	2.17E-18	533	533
APBB1IP	0.304758758	6.42E-13	5.87E-12	533	533
APBB1	-0.349864624	8.59E-17	1.25E-15	533	533
APOBEC	0.563878588	4.60E-46	4.22E-43	533	533
APOBEC	0.5175482	7.47E-38	2.32E-35	533	533
APOBEC	0.376030698	2.40E-19	4.75E-18	533	533
APOBEC	0.631536609	1.15E-60	1.16E-56	533	533
APOBEC	0.532298968	2.49E-40	1.14E-37	533	533
APOC1	0.392894849	4.05E-21	1.00E-19	533	533
APOC2	0.433093275	8.86E-26	3.92E-24	533	533
APOH	0.358297457	1.37E-17	2.20E-16	533	445
APOL1	0.445638582	2.29E-27	1.24E-25	533	533
APOL2	0.40921093	6.19E-23	1.93E-21	533	533
APOL6	0.466493989	3.72E-30	3.02E-28	533	533
APOLD1	-0.30637688	4.78E-13	4.46E-12	533	533
APP	-0.30465711	6.54E-13	5.96E-12	533	533
AQP7	-0.352310982	5.07E-17	7.58E-16	533	530
AQP9	0.399394226	7.87E-22	2.14E-20	533	533
ARC	-0.319769794	3.89E-14	4.13E-13	533	533
ARFGAP	-0.310314257	2.32E-13	2.25E-12	533	533
ARHGAP	0.420477249	3.01E-24	1.10E-22	533	533
ARHGAP	0.359698997	1.00E-17	1.63E-16	533	533
ARHGAP	-0.336646375	1.37E-15	1.73E-14	533	533
ARHGAP	0.416769771	8.24E-24	2.86E-22	533	533
ARHGAP	-0.354618573	3.07E-17	4.73E-16	533	533
ARHGAP	-0.349972118	8.40E-17	1.22E-15	533	533
ARHGAP	-0.313433094	1.30E-13	1.29E-12	533	532
ARHGAP	0.442502393	5.79E-27	3.04E-25	533	533
ARHGEF	-0.307286067	4.05E-13	3.81E-12	533	533
ARHGEF	-0.331817723	3.64E-15	4.42E-14	533	533
ARL11	0.411981006	2.97E-23	9.61E-22	533	533
ARL4C	0.422983942	1.51E-24	5.80E-23	533	533
ARNTL2	0.384598977	3.11E-20	6.90E-19	533	532
ARPC1B	0.459680172	3.18E-29	2.26E-27	533	533
ARPC2	0.414957687	1.34E-23	4.56E-22	533	533
ARPC3	0.407331149	1.01E-22	3.09E-21	533	533
ARPC5	0.372052365	6.08E-19	1.14E-17	533	533
ARRB2	0.319490864	4.10E-14	4.34E-13	533	533
ARRDC5	0.32882348	6.63E-15	7.81E-14	533	521
ARVCF	-0.305022656	6.12E-13	5.61E-12	533	533
ASB2	0.396045217	1.84E-21	4.79E-20	533	533
ASCL2	0.404070671	2.37E-22	6.81E-21	533	532
ASF1B	0.390134837	8.03E-21	1.93E-19	533	533
ASPHD1	0.305006114	6.14E-13	5.62E-12	533	532
ASPHD2	0.322208955	2.43E-14	2.65E-13	533	533
ASPM	0.523503991	7.72E-39	3.05E-36	533	533
ASTN2	-0.368721517	1.31E-18	2.36E-17	533	533
ATAD2	0.362771735	5.06E-18	8.51E-17	533	533
ATAD5	0.316553503	7.19E-14	7.37E-13	533	532
ATG12	0.341312256	5.23E-16	7.01E-15	533	533
ATIC	0.38382004	3.75E-20	8.23E-19	533	533
ATOH8	-0.308527609	3.23E-13	3.07E-12	533	533
ATP13A3	0.33578382	1.63E-15	2.04E-14	533	533
ATP1A3	0.36937662	1.13E-18	2.04E-17	533	528
ATP5S	-0.324118887	1.67E-14	1.86E-13	533	533
ATP6V1	-0.330735024	4.53E-15	5.43E-14	533	533
ATP6V1	0.314399341	1.08E-13	1.09E-12	533	533
ATP6V1	-0.317106603	6.47E-14	6.68E-13	533	533
ATP8B3	0.487344362	3.83E-33	5.23E-31	533	533
AUH	-0.401353224	4.77E-22	1.34E-20	533	533
AURKA	0.346456572	1.78E-16	2.49E-15	533	533
AURKB	0.448824708	8.83E-28	5.21E-26	533	533
AVPR1A	-0.325820208	1.20E-14	1.37E-13	533	531
AVPR2	-0.355271056	2.67E-17	4.15E-16	533	530
AXL	0.430368247	1.92E-25	8.14E-24	533	533
B3GALT	0.39061132	7.14E-21	1.72E-19	533	489
B3GNTL	0.342446959	4.13E-16	5.58E-15	533	533
BAG1	-0.513825444	3.02E-37	8.02E-35	533	533
BAHD1	-0.469376487	1.48E-30	1.29E-28	533	533
BAIAP3	-0.349598921	9.10E-17	1.32E-15	533	533
BAK1	0.307411234	3.96E-13	3.73E-12	533	533
BAP1	-0.305475117	5.64E-13	5.19E-12	533	533
BASP1	0.460542149	2.43E-29	1.76E-27	533	533
BAT2L1	-0.392778248	4.17E-21	1.03E-19	533	533
BATF2	0.367287727	1.82E-18	3.22E-17	533	533
BATF	0.454845275	1.42E-28	9.20E-27	533	533
BBS1	-0.337307085	1.20E-15	1.52E-14	533	533
BCAM	-0.317666931	5.82E-14	6.04E-13	533	533
BCAR1	-0.329337411	5.99E-15	7.08E-14	533	533
BCL11B	0.481316768	2.94E-32	3.61E-30	533	533
BCL2A1	0.45000909	6.18E-28	3.71E-26	533	533
BCL2L10	-0.328230135	7.46E-15	8.74E-14	533	471
BCL2L14	0.405198824	1.77E-22	5.19E-21	533	511
BCL2L2	-0.474925918	2.44E-31	2.49E-29	533	533
BCORL2	0.304370664	6.89E-13	6.27E-12	533	326
BCR	-0.384363911	3.29E-20	7.28E-19	533	533
BDH2	-0.325604435	1.25E-14	1.42E-13	533	533
BEND6	0.328972292	6.44E-15	7.60E-14	533	532
BET1	0.396618094	1.59E-21	4.16E-20	533	533
BEX1	-0.321400736	2.84E-14	3.07E-13	533	507
BEX4	-0.370401736	8.91E-19	1.63E-17	533	533
BEX5	-0.36756226	1.71E-18	3.03E-17	533	533
BHLHB9	-0.367054547	1.92E-18	3.38E-17	533	533
BICD1	0.327700225	8.28E-15	9.63E-14	533	533
BID	0.378959868	1.20E-19	2.46E-18	533	533
BIN2	0.370585076	8.54E-19	1.57E-17	533	533
BIRC3	0.540608466	8.84E-42	5.57E-39	533	533
BIRC5	0.441428017	7.94E-27	4.06E-25	533	533
BLM	0.438464794	1.89E-26	9.21E-25	533	532
BMP1	0.312371093	1.58E-13	1.56E-12	533	533
BNC2	0.302203225	1.02E-12	9.08E-12	533	533
BOC	-0.32131707	2.89E-14	3.11E-13	533	533
BRD3	-0.394626263	2.63E-21	6.76E-20	533	533
BRF1	-0.37372527	4.12E-19	7.90E-18	533	533
BRIP1	0.320339314	3.49E-14	3.73E-13	533	532
BRMS1L	-0.446464012	1.79E-27	9.92E-26	533	533
BSPRY	-0.450001378	6.20E-28	3.71E-26	533	531
BST2	0.311831767	1.75E-13	1.72E-12	533	533
BTBD11	0.413943816	1.76E-23	5.82E-22	533	533
BTBD9	-0.374848691	3.17E-19	6.19E-18	533	533
BTD	-0.319761853	3.90E-14	4.13E-13	533	533
BTK	0.453623366	2.06E-28	1.30E-26	533	533
BTLA	0.459680159	3.18E-29	2.26E-27	533	530
BTN3A1	0.406475844	1.27E-22	3.82E-21	533	533
BTN3A2	0.312459376	1.55E-13	1.54E-12	533	533
BTNL9	-0.306572826	4.62E-13	4.31E-12	533	533
BUB1B	0.422818136	1.58E-24	6.05E-23	533	532
BUB1	0.518900665	4.48E-38	1.46E-35	533	532
C10orf11	-0.327623108	8.41E-15	9.77E-14	533	533
C10orf41	-0.348069746	1.26E-16	1.81E-15	533	533
C10orf72	-0.310774684	2.13E-13	2.07E-12	533	533
C10orf81	0.331679465	3.75E-15	4.53E-14	533	498
C10orf99	0.475672041	1.91E-31	1.99E-29	533	492
C11orf45	0.424336816	1.04E-24	4.06E-23	533	533
C11orf73	0.301688114	1.12E-12	9.89E-12	533	533
C11orf75	0.388905434	1.09E-20	2.56E-19	533	533
C11orf82	0.393237868	3.72E-21	9.29E-20	533	532
C11orf86	0.372634905	5.32E-19	1.01E-17	533	485
C11orf92	-0.404889061	1.92E-22	5.61E-21	533	533
C11orf95	-0.339969653	6.91E-16	9.09E-15	533	533
C12orf32	0.49011016	1.48E-33	2.11E-31	533	533
C12orf35	0.302844795	9.08E-13	8.13E-12	533	533
C12orf45	0.357551524	1.62E-17	2.57E-16	533	533
C12orf48	0.360256862	8.87E-18	1.45E-16	533	532
C12orf49	-0.360401699	8.59E-18	1.41E-16	533	533
C13orf15	-0.303209725	8.50E-13	7.64E-12	533	533
C13orf18	0.388904316	1.09E-20	2.56E-19	533	533
C13orf34	0.494238518	3.54E-34	5.36E-32	533	531
